# Boldine Prevents Renal Alterations in Diabetic Rats

**DOI:** 10.1155/2013/593672

**Published:** 2013-12-12

**Authors:** Romina Hernández-Salinas, Alejandra Z. Vielma, Marlene N. Arismendi, Mauricio P. Boric, Juan C. Sáez, Victoria Velarde

**Affiliations:** ^1^Departamento de Fisiología, Pontificia Universidad Católica de Chile, Alameda Bernardo O'Higgins, No. 340, 6513677 Santiago, Chile; ^2^Instituto Milenio, Centro Interdisciplinario de Neurociencias de Valparaíso, Valparaíso, Chile

## Abstract

Diabetic nephropathy alters both structure and function of the kidney. These alterations are associated with increased levels of reactive oxygen species, matrix proteins, and proinflammatory molecules. Inflammation decreases gap junctional communication and increases hemichannel activity leading to increased membrane permeability and altering tissue homeostasis. Since current treatments for diabetic nephropathy do not prevent renal damage, we postulated an alternative treatment with boldine, an alkaloid obtained from boldo with antioxidant, anti-inflammatory, and hypoglycemic effects. Streptozotocin-induced diabetic and control rats were treated or not treated with boldine (50 mg/Kg/day) for ten weeks. In addition, mesangial cells were cultured under control conditions or in high glucose concentration plus proinflammatory cytokines, with or without boldine (100 *µ*mol/L). Boldine treatment in diabetic animals prevented the increase in glycemia, blood pressure, renal thiobarbituric acid reactive substances and the urinary protein/creatinine ratio. Boldine also reduced alterations in matrix proteins and markers of renal damage. In mesangial cells, boldine prevented the increase in oxidative stress, the decrease in gap junctional communication, and the increase in cell permeability due to connexin hemichannel activity induced by high glucose and proinflammatory cytokines but did not block gap junction channels. Thus boldine prevented both renal and cellular alterations and could be useful for preventing tissue damage in diabetic subjects.

## 1. Introduction

Diabetic nephropathy (DN) is considered a microvascular complication of diabetes. It is characterized by a chronic injury to renal tissue, mainly glomerular structures. DN is the major complication associated with type I diabetes mellitus, affecting approximately 30–40% of diabetic patients, and is the leading cause of end-stage renal disease [1].

There are several factors involved in the development of diabetic glomerulopathy but the main initiator of this disease is chronic hyperglycemia, which triggers nonenzymatic protein glycosylation and increased production of reactive oxygen species, increasing oxidative stress and favoring coagulation and fibrotic events [2]. Hyperglycemia also sensitizes the vessel wall of efferent arterioles to the action of vasoconstrictors, which, together with an increase in vascular permeability, lead to hyperfiltration. Also, in the progression of DN persistent proteinuria, glomerular hypertrophy, mesangial expansion, and tubulointerstitial fibrosis occur, which lead to partial or total loss of renal function[3, 4].

In diabetes and in different cell types cultured in high glucose concentration (from now and on named as high glucose), an increase in the inflammatory response is observed. This response is usually initiated by cytokine release and subsequent attraction of macrophages, important actors in the progression of DN [5, 6]. In this and other diseases where inflammation is present, the expression of connexins (Cxs), including Cx43, is altered and increased activity of Cx formed hemichannels is observed. The latter alterations have been linked to a reduction in gap junction activity (essential for intercellular communication) and have been considered a risk factor for certain diseases [7, 8].

In addition, TNF-*α* and IL-1*β*, two pro-inflammatory cytokines, reduce the activity of gap junctions [9, 10] and increase the activity of hemichannels in cortical astrocytes [11–13]. Even more, FGF-1, a growth factor that is elevated during diabetes, increases the activity of hemichannels formed by Cx43 [14, 15]. In cocultures of astrocytes and microglia, proinflammatory conditions decrease gap junctional communication and increase membrane permeability, effect attributed to an increased hemichannel activity [12]. Finally, it has been postulated that Cxs are increased in certain diseases that involve renal damage [16]. Consequently, Cx hemichannels have been proposed to be possible targets for new therapies.

As mentioned above, reactive oxygen species are increased in diabetes and oxidative stress induces opening of Cx43 hemichannels present in the cell membrane[17]. Interestingly, the effect of reducing agents, such as dithiothreitol, depends on the redox state of hemichannels. Dithiothreitol induces closure of oxidized channels but opens hemichannels under resting conditions that are preferentially in a reduced state [18]. In this sense, a blocking agent that closes hemichannels both in the oxidized and reduced state should provide a comparative advantage over those that only affect the activity of hemichannels by redox changes.

Currently, to prevent the progression of DN, patients are advised to strictly control their glycemia and blood pressure [19], but this treatment is not enough to improve renal function. In this paper, we propose the complementary use of boldine, an alkaloid obtained from *Peumus boldus*, to prevent the development of DN. This alkaloid has been proposed to be useful in the treatment of certain diseases involving free radicals such as atherosclerosis, ischemia-reperfusion, and inflammatory diseases, among others [20]. Furthermore, our preliminary studies have identified boldine as a Cx hemichannel blocker through a mechanism not yet identified but independent of its antioxidant property. Therefore, we propose that treatment with boldine could preserve a normal glomerular filtration and a tubular function during diabetes by blocking Cx hemichannels.

## 2. Methods

### 2.1. Boldine Purification

The hydrochloride form of boldine was prepared by Härting company (Santiago, Chile) and was obtained from boldo's bark (*Peumus boldus* Molina, Monimiaceae), following a procedure described by Urzúa and Acuña [21] with minor modifications. The purity of the alkaloid was checked by HPLC (high-performance liquid chromatography) analysis (99%) as described previously [22].

### 2.2. Animals and Experimental Procedures

Male Sprague-Dawley (SD) rats (180–200 g) bred in the bioterium of the Facultad de Ciencias Biológicas, Pontificia Universidad Católica de Chile, were used. Rats were separated in three groups: (1) diabetic rats that received a single injection of streptozotocin (45 mg/Kg body weight, IP); (2) diabetic rats additionally treated with boldine (diabetic + boldine) (50 mg/kg body weight in 1 mL, via gavage daily), for the last 10 weeks of experimentation; and (3) rats from the control group that only received the vehicle for the drug (1 mL 0.1 M citrate buffer, pH 4.5, by gavage daily). Animal protocols were approved by the Bioethical Committee of the Pontificia Universidad Católica de Chile and were in accordance with the Guide for the Care and Use of Laboratory Animals endorsed by the American Physiological Society. Animals were kept for 15 weeks in a room with an ambient temperature (22–25°C), 12 : 12 hours light : dark cycles, and food and water *ad libitum, *under the supervision of a veterinarian.

### 2.3. Renal Function

To determine physical parameters and renal function, animals were weighed and blood and urine samples were obtained every 5 weeks. To obtain urine samples animals were housed in individual metabolic cages for 24 h. During this time period they were deprived of food but had water *ad libitum*.

Blood samples were taken from the tail vein, under isoflurane anesthesia. To measure blood glucose Accutrend blood strips were used. Proteinuria was measured using the method of Bradford [23] and creatininuria was measured using a kinetic kit from Valtek Diagnostics Laboratory (Santiago, Chile).

At the end of each treatment, animals were sacrificed under anesthesia (ketamine 90 mg/kg/xylazine 10 mg/kg, IP). Kidneys were removed and cut into 5 mm slices. Part of the tissue was immediately frozen in liquid nitrogen for protein analysis and part was fixed in Bouin's solution for immunohistochemistry.

### 2.4. Measurement of Osmolarity

An Osmomat 030 osmometer (Gonotec GmbH, Berlin, Germany) was used to measure osmolarity in 50 *μ*L of either plasma or urine, according to the manufacturer's instructions.

### 2.5. TBARS Measurement

Levels of thiobarbituric acid reactive substances (TBARS) were estimated using the method described by Ramanathan et al. [24] with slight modifications. Kidney homogenate supernatants were mixed with SDS (8% w/v), TBA (0.8% w/v), and acetic acid (20% v/v) and heated for 60 min at 90°C. Precipitated material was removed by centrifugation, and the absorbance of the supernatant was determined at 532 nm. Levels of TBARS were calculated using a calibration curve with malondialdehyde (MDA, Sigma-Aldrich, Saint Louis, USA).

### 2.6. Blood Pressure Measurement

Blood pressure was measured in the tail of conscious rats using a computerized 20,390 CODA, Kent Science instrument (Torrington, CT), in a room at 25°C, with noise and light control. Rats were trained for one month before the experimental measurements.

### 2.7. Tissue Processing and Immunohistochemical Analysis

Kidney samples (5 mm thick) were fixed by immersion in Bouin's solution for 24 h at room temperature. Each tissue sample was dehydrated and embedded in Paraplast Plus (Monoject Scientific, St. Louis, MO) and serial sections 5 *μ*m thick were obtained with a rotary microtome and mounted on glass slides. Some of these sections were stained with hematoxylin-eosin (H&E) to evaluate tissue morphology and with periodic acid-Schiff (PAS) staining to identify glycogen and glycoproteins. Immunostaining was also performed using indirect immunoperoxidase technique to localize collagen III and *α*-smooth muscle actin (SMA). Briefly, tissue sections were deparaffinized, rehydrated, washed with 0.05 M phosphate buffered saline (PBS) pH 7.6, and incubated with primary antibody against collagen III (1 : 500) or *α*-SMA (1 : 500) overnight at 22°C. The secondary antibody and the corresponding peroxidase anti-peroxidase complex (PAP) were sequentially applied for 30 min, each at 22°C. The immunoperoxidase reaction was visualized by incubating sections in 0.1% (w/v) of 3,3′-diaminobenzidine and 0.03% hydrogen peroxide. The antibodies and PAP complex were diluted in Tris-phosphate-saline (TPS) buffer containing 0.25% Triton X-100 and 0.7% (w/v) *λ*-carrageenan. The sections were washed with PBS between incubations, contrasted with hematoxylin, and dehydrated. The images were acquired with a Nikon Eclipse E600 microscope equipped with a Nikon DXM1200 digital camera.

### 2.8. Cell Culture

The cell line MES-13, derived from mesangial cells (CRL-1927 from ATCC), was cultured for 48 h in a mixture of DMEM (containing either 5 mmol/L or 25 mmol/L glucose) and F-12 (2 : 1), giving a final glucose concentration of 7.5 mmol/L for control and 20 mmol/L for high glucose, respectively. These media were supplemented with 10% FBS, 100 U/mL penicillin, 100 *μ*g/mL streptomycin, and 0.025 *μ*g/mL Fungizone (amphotericin B). To the high glucose medium, TNF-*α* (10 ng/mL) and IL-1*β* (10 ng/mL) were added before the last 6 h of culture to mimic a diabetic environment. Boldine (100 *μ*mol/L) was added during the last 24 h of treatment.

### 2.9. Dye Uptake and Fluorescence Time-Lapse Imaging

MES-13 cells were seeded on glass coverslips and covered with a normal glucose medium. When cells reached confluence, they were washed twice in PBS, pH 7.4, and then bathed with Locke's recording saline solution (in mmol/L: 154 NaCl, 5.4 KCl, 2.3 CaCl_2_, and 5 HEPES, pH 7.4) with 5 *μ*mol/L ethidium (Etd) bromide. Cells cultured on glass coverslips were placed on an upright Olympus microscope BX 51W1I with water immersion objectives. Changes in fluorescence were monitored using a digital camera (12 bits, Qimaging, Burnaby, BC, Canada) and the program of acquisition and image analysis was METAFLUOR software (Universal Imaging Co., Downingtown, PA, USA). Fluorescence was recorded every 30 seconds for 10 min at room temperature (20–22°C). In the analysis, the regions of interest were placed over the cell nuclei selected at random and these regions were background subtracted. Average slope values were compared using GraphPad Prism software.

### 2.10. Dye Coupling

MES-13 cells were seeded on glass coverslips, covered with normal glucose medium, and the functional state of gap junctions was tested by evaluating the transfer of Etd from one microinjected cell into neighboring cells. Cells were placed in a recording saline solution (in mol/L: 154 NaCl, 5.4 KCl, 2.3 CaCl_2_, 5 HEPES, and 5 glucose, pH 7.4) and cultures were observed using an inverted microscope equipped with xenon arc lamp and a Nikon B filter (excitation wavelength: 450–490 nm, emission wavelength: above 520 nm). Etd (25 mmol/L) was microinjected through a glass microelectrode. Two minutes after injection, cells were observed to determine whether dye transfer occurred. The incidence of coupling corresponds to the percentage of cases in which dye spread occurred to more than one neighbor cell. The coupling index was calculated as the average number of cells to which the dye had spread divided by the number of positive cases. In all experiments, the intercellular coupling was tested by injecting a minimum of 10 cells.

### 2.11. Electrophoresis and Western Blot Analysis

To determine relative levels of Cx43, MES-13 cells were lysed in RIPA with protease inhibitors (1 mg/mL aminocaproic acid, 1 mg/mL benzamidine, 0.2 mg/mL SBTI, and PMSF 3 mmol/L) and phosphatase inhibitors (0.012 mg/mL sodium orthovanadate, 4.46 mg/mL sodium pyrophosphate, and 4.2 mg/mL sodium fluoride). Lysates were centrifuged and supernatants were collected for Western blot analysis. Protein concentrations were determined by the method of Lowry et al. [25]. Protein samples (50 *μ*g) from cells cultured under different treatments were separated by electrophoresis in 10% SDS-polyacrylamide gel (SDS-PAGE) and in one lane a sample of prestained molecular weight markers (MW) was resolved. Proteins were transferred to a 0.45 *μ*m PVDF membrane, which was blocked at room temperature with Tris pH 7.4/5% skim milk (w/v)/BSA 1% (w/v). Then, the PVDF membrane was incubated overnight at 4°C with anti-Cx43 (monoclonal, Zymed; 1 : 500), followed by incubation with rabbit secondary antibody conjugated to peroxidase (polyclonal, Santa Cruz Biotechnology; 1 : 1000) for 1 h. Then membrane was stripped and reblotted with antibody anti T-ERK (polyclonal, Santa Cruz Biotechnology, 1 : 4000) as loading control, following the same procedure as before.

Immunoreactive bands were visualized using a chemiluminescent reagent (Western Lightning, Perkin Elmer) according to the procedure described by the supplier and Kodak films X-LS. The bands detected were digitized and subjected to densitometric analysis using the program Image J (NIH, Washington, DC, USA).

### 2.12. Statistical Analysis

Data sets obtained from different groups of rats or cells, under the same condition, were compared with each other by one-way analysis of variance (ANOVA) followed by a Bonferroni's post hoc test. Differences were considered significant if *P* ≤ 0.05. The analyses were performed with GraphPad Prism 5 software for Windows (1992–2007, GraphPad Software).

## 3. Results

### 3.1. Boldine Prevents Hyperglycemia and Hypertension in Diabetic Rats

In order to establish a diabetic model and to study how it is affected by treatment with boldine, body weight, mean blood pressure, blood glucose, urine volume, osmolality, and lipid peroxidation were measured in plasma and urine of each rat from the three above mentioned groups.

Since an increased level of plasma glucose is the main feature in diabetes, this parameter was evaluated periodically. Plasma glucose values from all groups of animals at the end of the experiment are summarized in Table 1. The control group (C) had a normal glycemia, whereas the glycemia of the diabetic group (D) was significantly higher. Interestingly, the diabetic group treated with boldine (DB) had a glycemia lower than that of the diabetic group, not significantly different from the control group.

It is well documented that animals with experimental type I diabetes lose weight over time due to the loss of muscle and adipose tissue. At the end of the experiment, rats from both diabetic groups (D and DB) weighted significantly less than animals of group C with no significant differences within the two diabetic groups (Table 1).

Total urinary volume in a 24 h collection was significantly increased in the diabetic rats as compared to the control group. Interestingly, in the diabetic group treated with boldine the urinary volume was slightly reduced compared to the values of the diabetic group (Table 1).

Since high concentrations of glucose were detected in blood and urine from diabetic rats, we evaluated whether there were changes in osmolality. All groups showed similar values in plasma osmolality and total osmoles excreted in the urine in 24 h (Table 1).

In diabetes, oxidized lipids are increased due to an increase in oxidative stress and dyslipidemia [5]. TBARS values in both plasma and urine were significantly higher in D and DB groups when compared with the control groups (Table 1). Interestingly, boldine, which has a potent antioxidant capacity, did not modify TBARS values in these two fluids as evidenced when DB was compared to D.

One of the alterations that frequently occurs as a result of, or associated with, diabetes is hypertension. For this reason, mean blood pressure was measured in conscious rats. Control rats and rats treated with boldine showed normal blood pressure (Table 1), whereas blood pressure was significantly increased in the diabetic group when compared to control. Interestingly, group DB showed a blood pressure similar to control animals (Table 1).

### 3.2. Boldine Prevents Kidney Damage in Diabetic Rats

**Figure 1:**
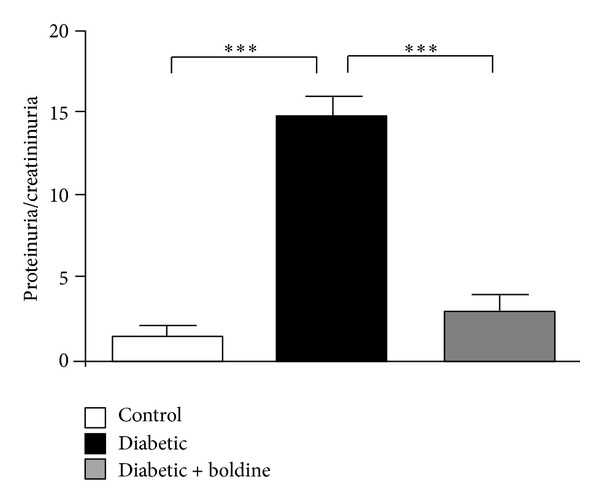
Boldine prevents kidney damage in diabetic rats. The graph shows the ratio of proteinuria/creatininuria in control (white bar), diabetic (black bar), and diabetic rats treated with 50 mg/Kg/day boldine (grey bar). Each bar represents the mean value ± SEM of 5 different animals. ***P ≤ 0.001, using the one-way ANOVA test followed by Bonferroni's post hoc test.

Diabetes, and in particular hyperglycemia, is a risk factor for renal function characterized by dysfunction in glomerular filtration rate (GFR). To evaluate renal function, the ratio between urinary proteins and urinary creatinine in the different groups was evaluated. Diabetic rats had significantly higher ratio (14.8 ± 1.2) when compared to control animals (1.7 ± 0.4), whereas diabetic rats treated with boldine showed values that were not significantly different from control (3.4 ± 1.0) but were significantly lower than group D (Figure 1).

### 3.3. Boldine Prevents Renal Morphological Changes in Diabetic Rats

**Figure 2:**
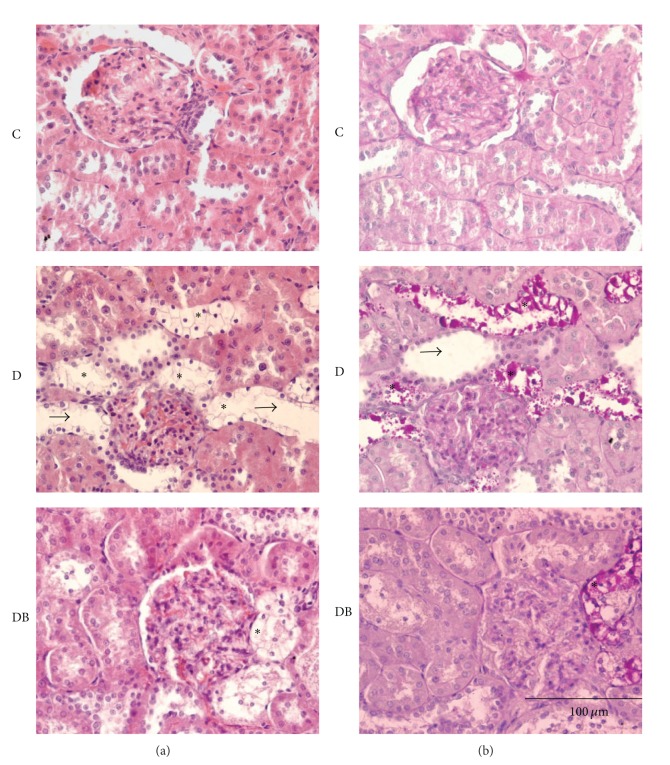
Boldine prevents the structural alterations in kidneys from diabetic rats. Serial kidney sections (5 *μ*m) were either stained with (a) hematoxylin and eosin or (b) with PAS staining. The top panels correspond to kidney sections from control rats (C), the middle panels are from diabetic rats (D), and the bottom panels are from diabetic rats treated with 50 mg/Kg/day boldine (DB). *: indicate clear cells; arrowheads indicate dilated tubules. Note close spatial correspondence between clear cells and PAS staining. Each picture is representative of at least 5 different animals.

In diabetes, excess blood glucose triggers the increase in oxidative stress producing a homeostatic imbalance at the cellular level that alters the renal histology. The structure of kidneys was altered in group D, as evaluated by H&E staining, which demonstrated dilated tubules (arrow head), increased interstitial matrix, and thickening of basement membrane in the glomerulus as compared to control (Figures 2(a) and 2(b)). In addition, clear cells (asterisks) were present in distal tubules. These alterations were largely absent in kidneys from group DB, in which changes were restricted mainly to the presence of few clear cells (Figure 2(a)). To determine the content of these clear cells we performed PAS staining that labels carbohydrates with an intense pink color. Both D and DB groups were positive for this staining (Figure 2(b)), and labeling colocalized with clear cells detected in H&E staining. PAS staining was abundant in group D, very infrequent in group DB, and absent in group C (Figure 2, middle, bottom, and top, panels resp.).

### 3.4. Boldine Reduces Protein Markers of Renal Damage in Diabetic Rats

**Figure 3:**
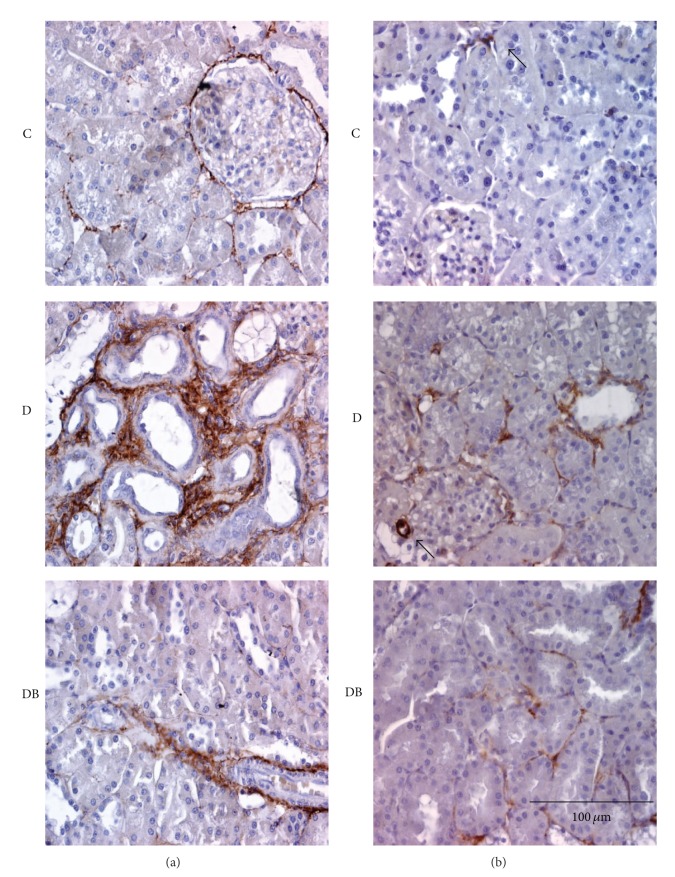
Boldine decreases markers of kidney damage in diabetic rats. Panels show immunohistochemistry for collagen III (a) and *α*-smooth muscle actin (SMA) (b). Top panels show fields of kidney sections from control (C) rats. Central panels show fields of kidney sections from diabetic rats (D) and the bottom panels are from diabetic rats treated with 50 mg/Kg/day boldine (DB). Each picture is representative of at least 5 different animals. Arrowheads show staining in vascular tissue.

In diabetes, an increase in kidney interstitial matrix proteins is observed [26]. Therefore, we evaluated if boldine prevents this modification in diabetic rats. Sections from group C, stained for collagen III, showed a discreet brown staining, which was limited mainly to the basal lamina and blood vessels (Figure 3(a), top panel). In contrast, group D showed a large increase in brown staining located mainly in peritubular areas (Figure 3(a), middle panel). Interestingly, group DB showed a staining very similar to that detected in control animals (Figure 3(a), bottom panel).

Presence of myofibroblasts, characterized by the reactivity to *α*-SMA, has been detected in the interstitium of diabetic kidneys [27, 28]. As expected, kidneys from group C only presented *α*-actin from smooth muscle (*α*-SMA) staining in blood vessels, more specifically in smooth muscle cells (Figure 3(b), top panel, arrowhead). However, kidneys from group D, in addition to the vascular labeling, showed a strong labeling in peritubular areas (Figure 3(b), middle panel). This reactivity was clearly reduced in the DB group (Figure 3(b), bottom panel).

### 3.5. Boldine Prevents the Increase in Lipid Peroxidation in Kidneys from Diabetic Rats and in Cells in a Diabetic Environment

**Figure 4:**
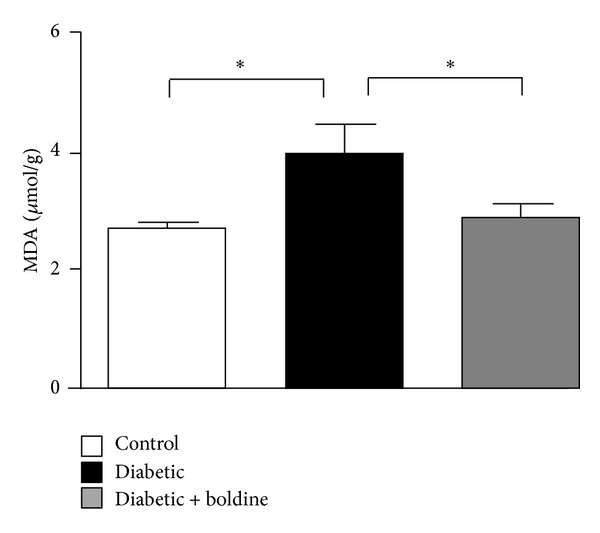
Boldine prevents lipid peroxidation in kidneys from diabetic rats. Graph showing levels of TBARS (MDA) in kidneys from control rats (white bar), diabetic rats (black bar), and diabetic rats treated with 50 mg/Kg/day boldine (grey bar). Values are expressed as *μ*mol of MDA per g of renal tissue. Each bar represents the mean value ± SEM of tissue from 7 independent animals. *P ≤ 0.05, using the one-way ANOVA test followed by Bonferroni's post hoc test.

**Figure 5:**
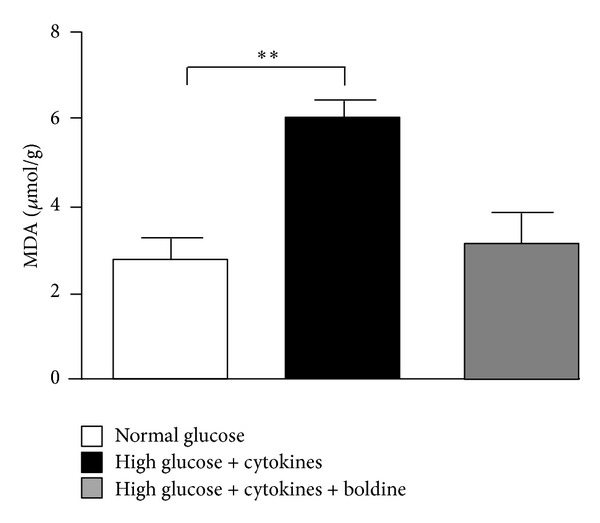
Boldine prevents lipid peroxidation in mesangial cells grown in high glucose and pro-inflammatory cytokines. Graph showing levels of TBARS (MDA) in the culture medium of mesangial cells cultured for 48 h, under normal glucose (white bar), high glucose + cytokines (TNF-*α* (10 ng/mL) and IL-1*β* (10 ng/mL)) (black bar), or high glucose + cytokines treated with 100 *μ*M boldine during the last 24 h (grey bar). Values are expressed as *μ*mol of MDA per g of protein in the culture medium. Each bar represents the mean value ± SEM of 4 independent experiments. **P ≤ 0.01, using one-way ANOVA test followed by Bonferroni's post hoc test.

We also quantified TBARS in renal homogenates, as an indication of the tissue redox status. TBARS from group D (4.1 ± 0.4 *μ*mol/g of kidney) were significantly higher than those in control samples (2.7 ± 0.1 *μ*mol/g), whereas in group DB; TBARS were similar to control values (2.9 ± 0.2 *μ*mol/g) (Figure 4). Similarly, TBARS were measured in the supernatant of mesangial cells grown for 48 h in control medium or in high glucose medium plus pro-inflammatory cytokines, mimicking the diabetic environment. The content of TBARS in supernatant of cells grown in medium containing high glucose and pro-inflammatory cytokines was twice that found in cells cultured under control conditions (6.0 ± 0.5 versus 2.8 ± 0.6 *μ*mol/g protein) (Figure 5). However, the increase in TBARS was fully prevented in cells maintained in high glucose and pro-inflammatory cytokines treated with boldine for the last 24 h (Figure 5).

### 3.6. Boldine Prevents the Increase in Membrane Permeability of Cells Grown in a Diabetic Environment

**Figure 6:**
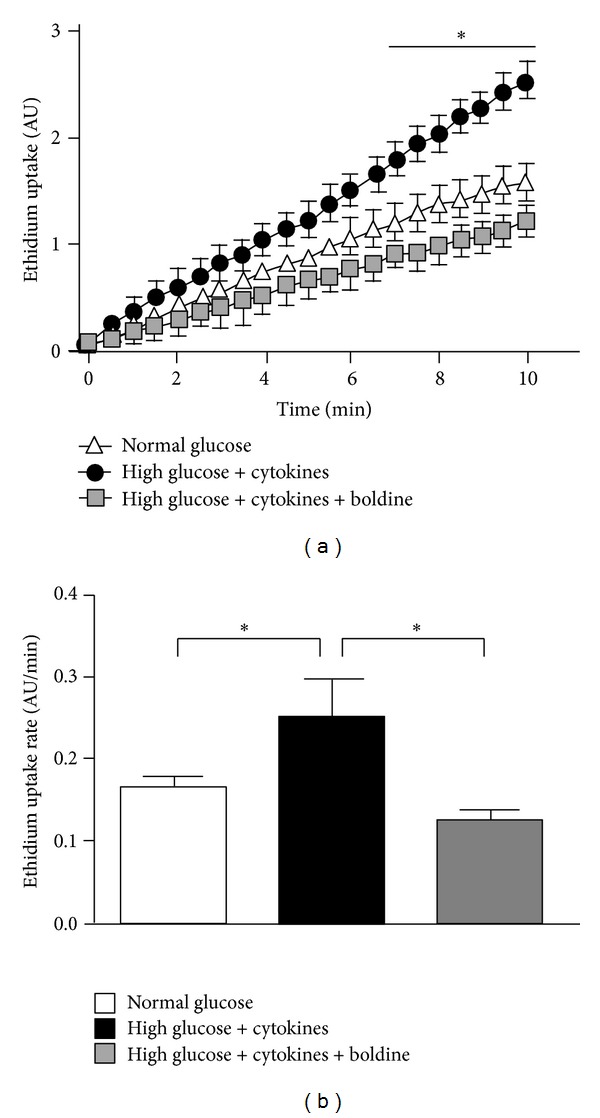
Boldine reduces the increase in Etd uptake induced by high glucose + cytokines in mesangial cells. (a) The graph shows time-lapse measurements of fluorescence intensity of Etd bound to nucleic acids of mesangial cells cultured for 48 h under normal glucose (white triangle), high glucose + cytokines (TNF-*α* (10 ng/mL) and IL-1*β* (10 ng/mL)) (black circles), and high glucose + cytokines treated with 100 *μ*M Boldine for the last 24 h (grey square). (b) Each bar represents the mean value of the rate of Etd uptake obtained from time-lapse curves. Mean ± SEM for 4 independent experiments. Normal glucose (White bar), high glucose + cytokine (black bar), or high glucose + cytokine with 100 *μ*M boldine (grey bar) for 24 h; *P ≤ 0.05 using one-way ANOVA test followed by Bonferroni's post hoc test.

Under resting conditions, Cx hemichannels of different cells in culture show a low open probability but in the presence of pro-inflammatory conditions they open even at negative membrane potential and in the presence of extracellular divalent cations [29–31], contributing to a deregulation in the electrochemical gradient across the cell membrane. In diabetes, pro-inflammatory cytokines are increased [10]. For this reason, it was expected that activity of renal Cx hemichannels could be increased. Since it is difficult to evaluate the activity of hemichannels in a tissue, we estimated the Cx hemichannel activity by determining the dye uptake rate from time-lapse measurements of Etd uptake in cultured mesangial cells (Figure 6(a)). The rate of Etd uptake (slope) of control cells incubated in normal glucose was 0.17 ± 0.01 AU/min, whereas cells grown in high glucose plus cytokines showed a slope of 0.25 ± 0.05 AU/min, which was significantly higher than that of control cells (Figure 6(b)). In cells incubated in high glucose + cytokines and treated with 100 *μ*mol/L boldine the rate of Etd uptake was not different from that of control cells (0.13 ± 0.01 AU/min).

### 3.7. Boldine Prevents the Reduction in Cell Coupling via Gap Junctions in Mesangial Cells Cultured in a Diabetic Environment

**Figure 7:**
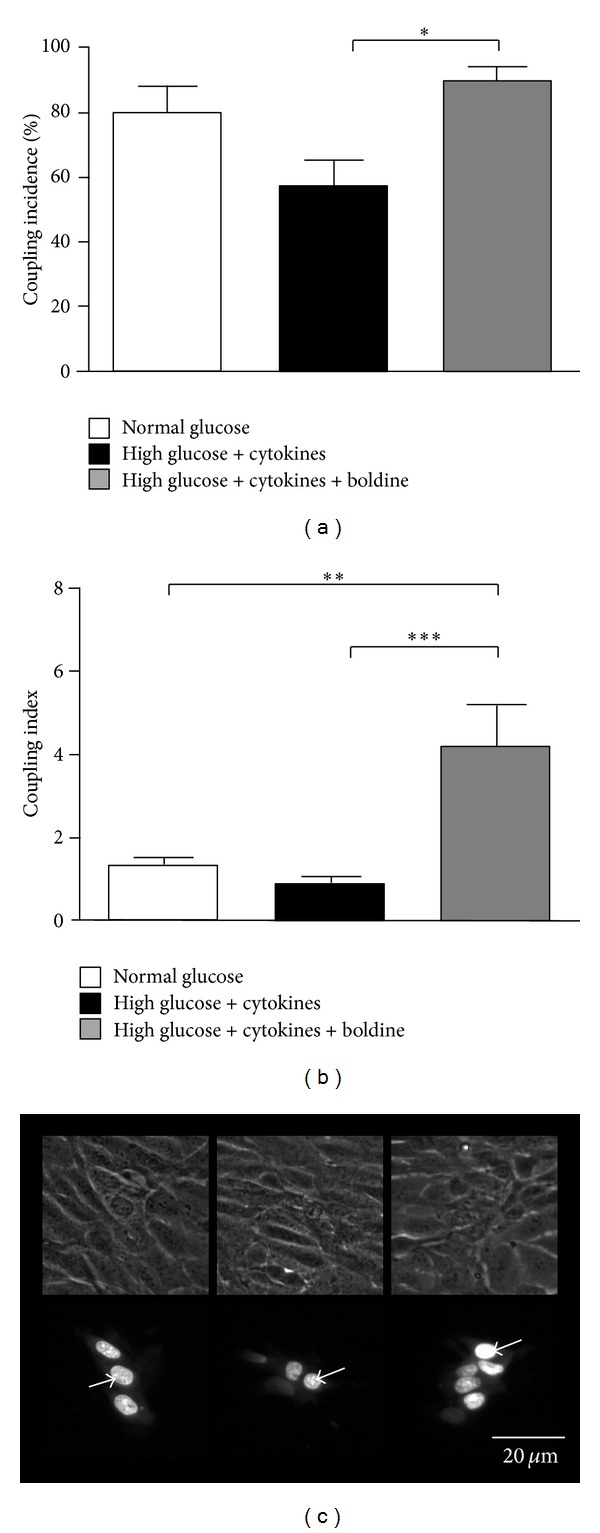
Boldine increases gap junctional coupling between mesangial cells. Coupling incidence (a) and coupling index (b) were evaluated in confluent mesangial cell cultures for 48 h, under different conditions using the dye (Ethidium bromide) coupling technique: normal glucose (white bar), high glucose + cytokine (TNF-*α* (10 ng/mL) and IL-1*β* (10 ng/mL)) (black bar), or high glucose + cytokine treated with 100 *μ*M boldine for the last 24 h (grey bar). Each bar represents the mean value ± SEM of 5 independent experiments. In each experiment the dye was microinjected into at least 10 cells. **P ≤ 0.01; ***P ≤ 0.001, using the one-way ANOVA test followed by Bonferroni's post hoc test. (c) Etd was microinjected into the brightest cell (arrow) and diffused to neighboring cells. Normal glucose (left bottom), high glucose + cytokine (middle bottom), and high glucose + cytokine and treated with 100 *μ*M boldine (right bottom) for 24 h. The top panels are the corresponding phase views. Scale bar = 20 *μ*m.

Gap junctions mediate direct intercellular communication of ions and small molecules including second messengers [32]. This type of intercellular communications is frequently reduced by pro-inflammatory conditions [12, 29–31]. Mesangial cells cultured for 48 h in high glucose plus cytokines showed a decreased coupling incidence of 57 ± 8% from a control value of 80 ± 8%. However, in cells incubated with 100 *μ*mol/L boldine for 24 h, the coupling incidence was 95 ± 3%, indicating that boldine reverted the reduction in coupling induced by these diabetic conditions (Figure 7(a)). In addition, in cells cultured in high glucose plus cytokines and boldine the number of coupled cells (coupling index) increased to 4.4 ± 0.7, compared to 1.3 ± 0.2 in cells grown in normal glucose and 0.9 ± 0.2 in cells grown in high glucose for 48 h (Figures 7(b) and 7(c)).

### 3.8. Boldine Increases the Relative Levels of Cx43 in Mesangial Cells Grown in High Glucose Plus Proinflammatory Cytokines

**Figure 8:**
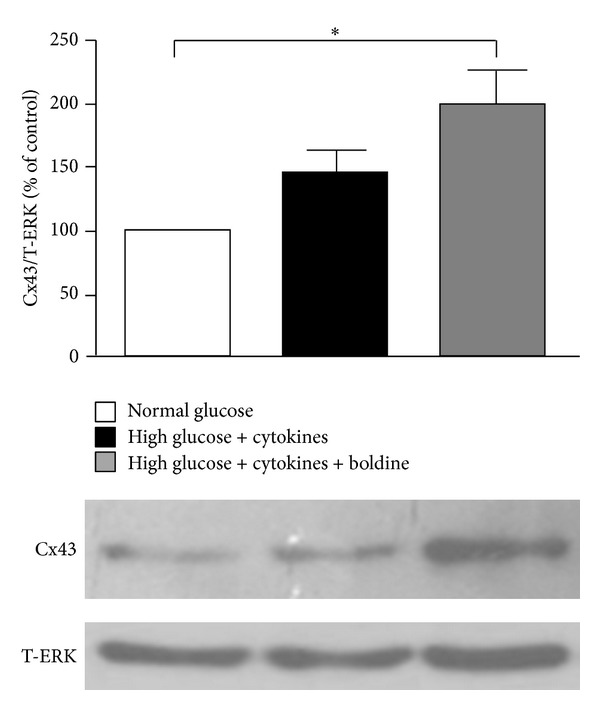
Boldine increases the relative Cx43 levels in mesangial cells grown in high glucose plus pro-inflammatory cytokines. Graph showing the relative levels of Cx43 under different conditions: normal glucose (white bar), high glucose + pro-inflammatory cytokines (TNF-*α* (10 ng/mL) and IL-1*β* (10 ng/mL)) (black bar), or high glucose + cytokines treated with 100 *μ*M boldine (grey bar) for 24 h. Each bar represents the mean value ± SEM of 4 independent experiments. *P ≤ 0.05, using the one-way ANOVA test followed by Bonferroni's post hoc test.

In inflammatory conditions, changes in the expression of Cxs have been found [33]. Cx43 is one of the most important gap junction subunits found in the glomerulus [34]. For this reason, the level of this protein was evaluated in mesangial cells cultured under diabetic conditions. In MES-13 cells maintained in a diabetic environment a slight but not significant increase in relative protein levels of Cx43 (143 ± 20%) was observed (Figure 8). Interestingly, in cells incubated in high glucose + cytokines in the presence of 100 *μ*mol/L boldine the increase in Cx43 levels was even higher (199 ± 26%) and statistically different from control (Figure 8).

## 4. Discussion

Previous studies have reported that boldine prevents the inflammatory response in rats [35] and treatment of diabetic rats with boldine reduces blood glucose levels [36] and decreases oxidative stress [37]. This study confirmed the hypoglycemic and antioxidant effects of boldine in diabetic rats; however, the most innovative findings of this research are the protective effect of boldine on renal parenchyma in diabetic rats and its inhibitory effect on Cx Hemichannels.

The reduction in glycemia observed in diabetic rats treated with boldine could be due to an inhibitory effect on *α*-glucosidase [38]. This enzyme, acts upon 1,4-alpha bonds, breaking down starch and disaccharides to glucose. In addition, boldine might reduce blood glucose by its action as an insulin sensitizer helping to increase glucose utilization in skeletal muscle [36] or by reducing glucose reabsorption in renal tubules.

The elevated blood pressure observed in diabetic rats could be due to vascular damage and renal damage caused by the increase in oxidative stress. The preclusion of an increase in blood pressure in the diabetic group treated with boldine is in agreement with this notion, since boldine has been postulated as a potent antioxidant [39]. The finding that boldine prevented the increased tissue TBARS levels but not urinary TBARS in diabetic rats was not expected. This observation might have at least two explanations. One of them is that, in diabetes, in the absence of boldine, TBARS could accumulate in the cell membrane of renal cells when compared to urine or plasma. Alternatively, it is possible that kidneys are capturing by reabsorption and accumulating into renal cells filtered TBARS generated in other organs, and for this reason it is easier to observe significant differences in kidney than in body fluids. In contrast, in the presence of boldine, the accumulation of TBARS in renal tissue is lower but they are still eliminated to the urine.

In addition to the effect in oxidative stress, there are several other ways by which boldine could prevent the increase in blood pressure. First, boldine might act as inhibitor of acetylcholinesterase [38]. If this is so, boldine could increase acetylcholine concentration at the vascular wall that in turn may contribute to the relaxation of blood vessels, and consequently, to the reduction in blood pressure. Second, boldine might prevent renal damage, which could help maintaining the normal balance of the vasoactive systems present in kidneys. Two major hormonal systems involved in blood pressure regulation are present in kidneys. They are the renin angiotensin system that induces vasoconstriction and water and salt retention and the kallikrein kinin system, which induces vasorelaxation and water and sodium excretion [19, 40]. The main enzymes of these systems are localized in tubular segments of the kidney. By preventing impairment in renal tubular epithelia, boldine might contribute favorably to maintain a normal balance of these two systems, and thus, a normal pressure is maintained in these animals. In support of this notion, in diabetic rats treated with boldine, proteinuria and creatininuria remained within normal ranges and morphological alterations in kidney, such as thickening of the glomerular basement membrane, tubular dilation, and accumulation of glycogen in the distal tubules, were largely prevented. Also a decrease in fibrosis and inflammatory cell infiltration were observed in the group of diabetic rats treated with boldine.

Although the kidney receives an important proportion of the cardiac output, this tissue is very sensitive to hypoxia, which produces a fast death of tubular cells, activating an inflammation response, characterized by macrophage infiltration and matrix accumulation. Therefore, boldine could also be protecting the renal tissue by preventing the hypoxia-induced damage. Several authors have postulated an important action of Cx43 in preconditioning [41]. In this sense, the increase in Cx43 in response to boldine could explain in part the protective effect of this alkaloid.

We propose that prevention of the damage is in part due to the blockade of hemichannels and increased gap junction activity. As it has been reported, inflammatory conditions lead to increased opening of hemichannels, promoting the loss in homeostasis and impaired cell function [12, 29–31, 42]. Our results show that mesangial cells kept in a diabetic environment presented an increase in hemichannel activity and a loss of cell communication via gap junctions, which was prevented by treatment with boldine, resulting in an increase in the number of coupled cells, an effect that is essential for the proper function of the kidney. The further increase in Cx43 protein levels in the presence of boldine could account for newly synthesized gap junctions and explain the increase in the number of coupled cells.

In conclusion, the alterations found in diabetic rats as hyperglycemia, hypertension, and renal damage were prevented in diabetic rats treated with boldine, suggesting that the harmful effects generated by increased oxidative stress and proinflammatory state in diabetes were diminished with boldine, not only by its antioxidant action but also due to the maintenance of cellular homeostasis by preventing the opening of hemichannels and keeping cells coupled to each other.

## Figures and Tables

**Table 1:** Pathophysiological status of experimental animals.

	Control (C)	Diabetic (D)	Control boldine (CB)	Diabetic boldine (DB)
Glycemia (mg/dL)	141 ± 8	371 ± 74***	147 ± 8	253 ± 31^†^
Weight (g)	530 ± 28	223 ± 25***	458 ± 22	235 ± 19***
Urine volume (mL)	16 ± 2	28 ± 5*	15 ± 3	24 ± 3
Plasma osmolality (mOsmol/Kg)	290 ± 4	310 ± 8	300 ± 6	300 ± 8
Urine omoles (in 24 h)	17 ± 1	23 ± 4	15 ± 1	24 ± 3
MDA plasma (nM)	50 ± 6	117 ± 9**	57 ± 7	121 ± 22**
MDA urine (pmoles in 24 h)	167 ± 20	842 ± 218**	127 ± 15	764 ± 97**
Mean blood pressure (mmHg)	116 ± 5	147 ± 12*	109 ± 9	119 ± 5^†^

Parameters observed in control (C), diabetic (D), and control or diabetic rats treated with 50 mg/Kg boldine (CB and DB, resp.) measured at the end of the experiment. Values represent the average ± SEM of at least 5 animals per group. *P ≤ 0.05; **P ≤ 0.01; ***P ≤ 0.001 compared to control; ^†^ *P* ≤ 0.05 compared to diabetic, using one-way ANOVA followed by Bonferroni's post hoc test.
